# Material Characterization of AISI 316L Flexure Pivot Bearings Fabricated by Additive Manufacturing

**DOI:** 10.3390/ma12152426

**Published:** 2019-07-30

**Authors:** Mirko Riede, Matthias Knoll, Christoph Wilsnack, Samira Gruber, Alba Alegre Cubillo, Christian Melzer, Ana Brandão, Laurent Pambaguian, André Seidel, Elena Lopez, Frank Brueckner, Christoph Leyens

**Affiliations:** 1 Fraunhofer Institute for Material and Beam Technology IWS, Winterbergstraße 28, 01277 Dresden, Germany; 2 Institute of Materials Science, Technische Universität Dresden, Helmholtzstr. 7, 01069 Dresden, Germany; 3 RUAG Space Germany GmbH, Am Glaswerk 6, 01640 Coswig, Germany; 4 European Space Research and Technology Centre—ESTEC, 2201 AZ Noordwijk, The Netherlands; 5 Department of Engineering Sciences and Mathematics, Luleå University of Technology, 971 87 Luleå, Sweden

**Keywords:** additive manufacturing, laser metal deposition, flexure pivot, space application

## Abstract

Recently, additive manufacturing (AM) by laser metal deposition (LMD) has become a key technology for fabricating highly complex parts without any support structures. Compared to the well-known powder bed fusion process, LMD enhances manufacturing possibilities to overcome AM-specific challenges such as process inherent porosity, minor build rates, and limited part size. Moreover, the advantages aforementioned combined with conventional machining enable novel manufacturing approaches in various fields of applications. Within this contribution, the additive manufacturing of filigree flexure pivots using 316L-Si by means of LMD with powder is presented. Frictionless flexure pivot bearings are used in space mechanisms that require high reliability, accuracy, and technical cleanliness. As a contribution to part qualification, the manufacturing process, powder material, and fabricated specimens were investigated in a comprehensive manner. Due to its major impact on the process, the chemical powder composition was characterized in detail by energy dispersive X-ray spectroscopy (EDX) and inductively coupled plasma optical emission spectrometry (ICP-OES). Moreover, a profound characterization of the powder morphology and flowability was carried out using scanning electron microscopy (SEM) and novel rheological investigation techniques. Furthermore, quantitative image analysis, mechanical testing, laser scanning microscopy, and 3D shape measurement of manufactured specimens were conducted. As a result, the gained knowledge was applied for the AM-specific redesign of the flexure pivot. Finally, a qualified flexure pivot has been manufactured in a hybrid manner to subsequently ensure its long-term durability in a lifetime test bench.

## 1. Introduction

### 1.1. Motivation

**Figure 1:**
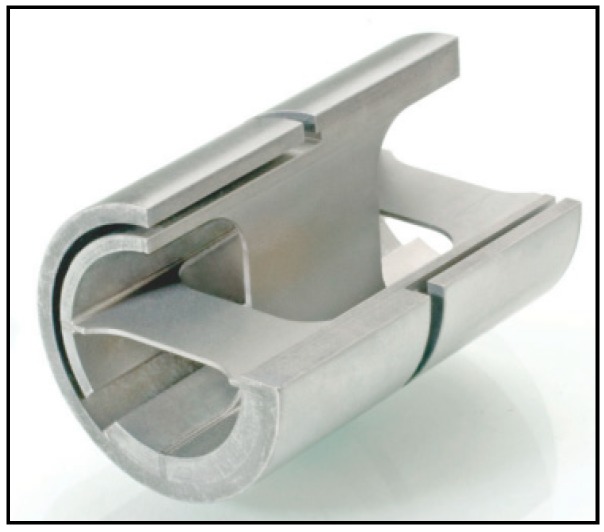
Brazed or welded stainless steel cantilevered pivot bearing of the Riverhawk Company.

Flexural pivots, flexible element hinges or compliant mechanisms are devices that connect or transmit loads between two components by elastically deflecting in an allowable range [1]. They are used in industrial or space applications, where high reliability, accuracy, and demanding requirements to contamination apply. Further advantages are, e.g., no need for assembly due to single-piece manufacturing, no friction losses, no need for lubrication due to lack of joints, and no wear by erosion due to lack of physical contact between kinematic pairs [2,3]. In space applications, flexible hinges are most commonly used in instrument mechanisms, e.g., for precise rotation of mirrors in a limited angular range, but also for the deployment of appendages and deployable booms, as so called collapsible flexible hinges. The state-of-the-art flexible hinges in space applications are mainly used in a rather narrow angular range, typically below +/− 10° for a very high number of cycles. Critical aspects are material selection, manufacturing accuracy, as well as fatigue behavior. Typical materials used for space applications are aluminium alloys (2024 T8, 6061 T6, 7075 T73, etc.), titanium alloys (especially Ti-6Al-4V), stainless steel (300 or 400 series including 440C for ball bearings, 15-5 PH with H1000 and above, 17-7 PH CH900, etc.), and nickel alloys (Inconel 718, etc.) [4,5]. In case of collapsible flexible hinges, carbon fiber-reinforced plastics (CFRP) or shape memory alloys (SMA) are also used to achieve a large angle of rotation (typically > 90°); however, these hinges are designed only for very few cycles (typ. 10–50). Conventional manufacturing methods for metallic flexure pivots are wire electrodischarge machining (WEDM) [6], laser cutting, or end-milling [2]. Commercially available hinges are, for instance, used in series mechanisms for lithography applications with very demanding cleanliness, reliability, fatigue, and repeatability requirements. An example of such a commercially available hinge is shown in Figure 1.

### 1.2. Material and Process Selection

**Figure 2:**
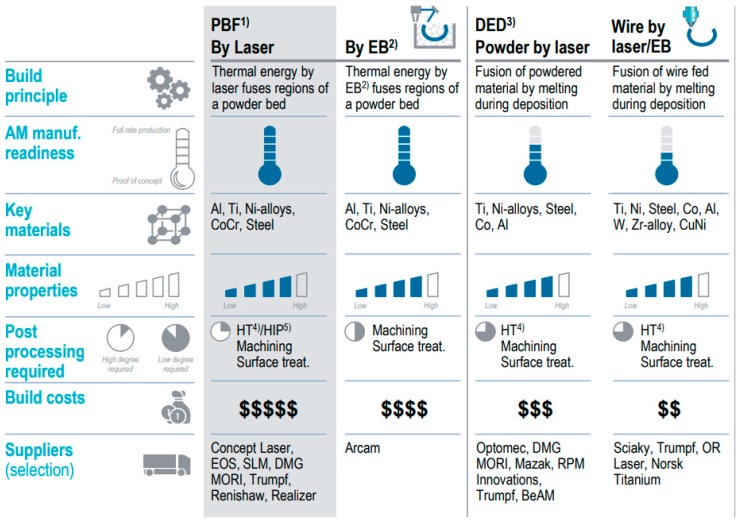
Additive manufacturing methods considered in process selection (extract of Roland Berger Report 2017).

For space applications, usually one-off custom designed solutions are needed. Additive manufacturing methods are promising in fabricating single piece metal flexural pivots with complex geometry (see Figure 2) [7].

For small and filigree additive manufactured components, laser powder bed fusion (LPBF) is usually considered due to the freedom of design and short lead times [4]. However, even this innovative technology has manufacturing constraints, such as the need of support structures or high build-up times, which affect the cost efficiency as well as process stability. In contrast to powder bed processes or competing direct methods (e.g., WAAM, EBAM), additive manufacturing via powder LMD provides

**Figure 3:**
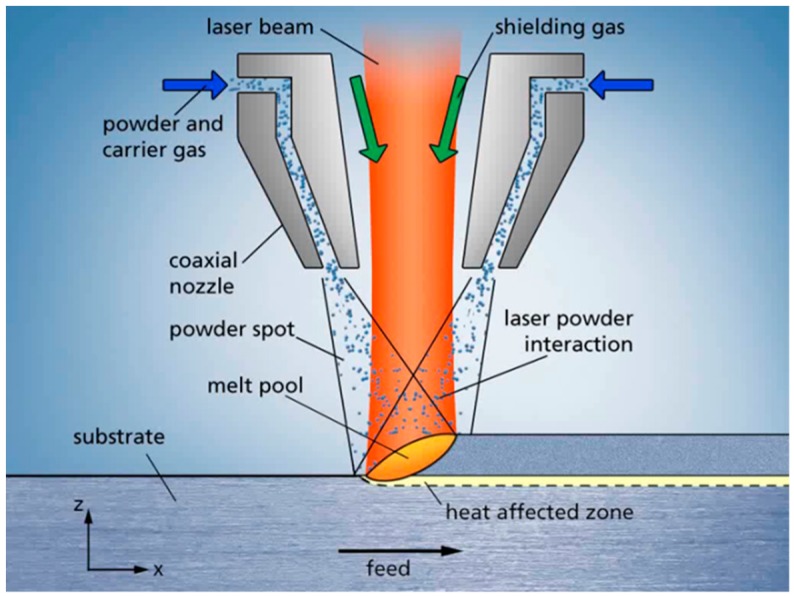
Powder laser metal deposition (LMD) process.

- supportless manufacturing (confer PBF),
- high productivity (confer PBF),
- high flexibility due to local shielding (confer PBF, EBAM),
- precise energy input—beneficial microstructure (confer WAAM, EBAM),
- low porosity HIP not needed (confer PBF) and
- hybrid manufacturing in one machine (confer PBF, EBAM),

These factors make this technology suitable for the realization of high-performance component designs. Besides, a further advantage of LMD is that conventionally manufactured semifinished parts can be used, adding new features via LMD. This approach decreases the manufacturing time and potentiates the advantages of hybrid AM processes. Therefore, powder LMD has been established in several branches, e.g., aerospace, medical, or tooling industries for the production of components for jet engines, implants, or drilling tools [8]. To deposit material on a substrate, the powder material is blown into the process zone by a nozzle, partially preheated in the laser beam and finally reabsorbed in the laser as illustrated in Figure 3.

This contribution aims at showing that LMD is suited for manufacturing of thin walled, flexible elements when combined with subtractive conventional machining. The end-to-end process chain was first designed and then tested to ensure qualification of all process steps to meet the specifications of the flexural pivot.

The material selected for this paper is stainless steel 316L-Si, as it provides high strength and is successfully approved by many space missions. Some of the 316L-Si properties are relatively high hardness and toughness, high corrosion resistance, and a beneficial machinability. It can be highly polished, which is an important aspect to reach the sophisticated demands of the highly loaded flexure pivot surfaces to eliminate cracks on the surface and thereby prevent failure by crack propagation. In contrast to titanium alloys, the price of stainless steel powder is much lower with good processability of the semifinished parts. Titanium alloys exhibit low thermal conductivity in conjunction with high strength leading to high abrasion of the machining tools in comparison to 316L-Si [9]. Additionally, the sensitivity of titanium alloys to oxygen at higher temperatures complicates the LMD process, as a global inert atmosphere is needed. Furthermore, steel 316L-Si can be processed with a local shielding gas atmosphere and it is a well-established material in additive manufacturing processes. For postprocessing, jig grinding was chosen to minimize mechanical loads on the filigree hinge while reaching high geometrical accuracy and low roughness.

## 2. Experimental Procedure

### 2.1. Parameter Development

Experimental trials based on simplified geometry features accompanied by metallographic investigations were conducted to sort out parameter sets suitable in terms of defect free build-ups and geometrical requirements (Table 1).

A disk powder feeder with a groove depth of 0.6 mm and a width of 5.0 mm was used. The parameter development was realized on the basis of single tracks and is composed of the following four steps:

- material processing,
- width adjustment,
- height adjustment and
- multilayer build-ups

The material processability was confirmed by producing single tracks with varying parameter sets. As recommended the powder was dried (3 h, 120 °C). The boundary condition for the second step was a desired track width of ~1 mm, chosen for reasons of stiffness, process stability, and machining time. Given that the track width primarily correlates with the laser power, several single tracks were welded and investigated with nonvarying parameters except for the laser power, which was decreased by 50 W track by track beginning at 600 W. A laser power of 550 W with a feed of 600 mm/min and a powder feed of 6 g/min delivered the required track width.

Next, the powder feed of the previous determined parameter set was varied to adjust the track height. The proposed track height to width ratio was 1/3 to 1/2 in order to guarantee a suitable accuracy as well as build-up rate. It was found that a powder feed of 4.16 g/min complied with the guideline.

**Figure 4:**
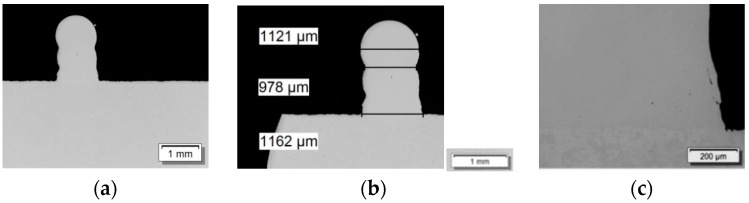
Cross section of multilayer build-up: (**a**) overview; (**b**) structure width; (**c**) detailed view of the defect free transition area.

Concluding, multilayer build-ups were realized using the developed parameter set in order to examine the steadiness of the process. The build-ups with the required height were metallographically investigated for cracks or pores and their size (Figure 4). The determined parameter sets were kept constant throughout the complete process development.

### 2.2. Test Sample Manufacturing

**Figure 5:**
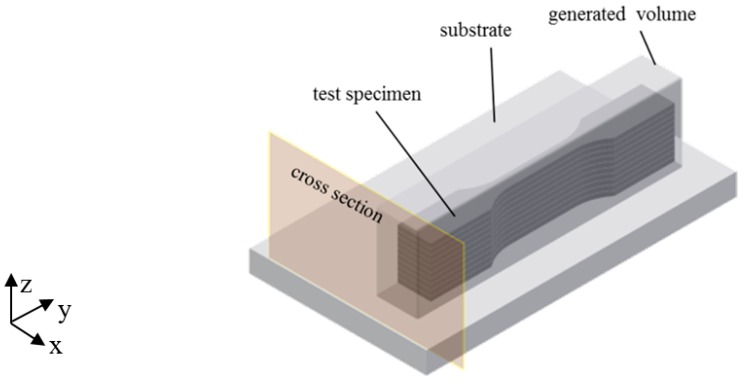
Solid block for flat tensile test specimens and metallographic witness sample.

**Figure 6:**
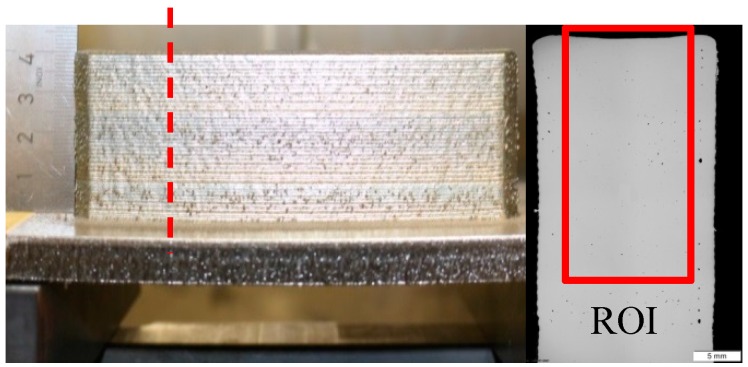
Material sample and the associated cross section.

To evaluate the material properties of the AM parts built with 316 L-Si and the powder-LMD process, material samples for density and tensile tests were manufactured from a solid block built with the following parameter-set (see Table 2 and Figure 5):

To homogenize thermal gradients associated with representative material properties, a scan strategy with 45° scan vectors in relation to the longitudinal material sample axes was chosen. In addition, the scan vectors were rotated by 90° after each layer. The generated material samples were heat treated at 420 °C for 1 h at an inert atmosphere to avoid excessive oxidation and then cooled down in the furnace to reduce the manufacturing process immanent residual stresses in the consolidated material. To avoid a grain boundary sensitization and a loss in oxidation resistance by heating up to temperatures higher than 450 °C, an annealing temperature of 420 °C was applied. After a holding time of 1 h, the samples were cooled down in the furnace. As can be seen in the metallographic investigation of the witness sample, only minor porosity and no cracks occurred in the test sample area (Figure 6). Larger pores are visible close to the surface outside the region of interest (abbreviation: ROI) due to the applied laser scanning strategy of filled tracks and contour and their insufficient overlapping.

Subsequently, the specimen cubes for the density test and the flat tensile strength specimen according to [10] were milled and then sectioned by wire electrical discharge machining (WEDM). Upon completion of this step, the material characterization was executed.

### 2.3. Powder and Material Characterization

Since the powder specifications play a major role in the laser-particle interaction within the additive manufacturing process, the build-up quality will be strongly affected by the powder material. Hence, the process stability as well as the quality of the flexure pivots will be ensured by means of a comprehensive power characterization, including: chemical composition, morphology, and rheology.

The used material for the build-up is the austenitic stainless steel powder Oerlikon Metcoclad 316L-Si which is similar to the AISI-Type 316L steel, modified only by an increased silicon level. The silicon acts as fluxing agent during the welding process and leads to a better deposition quality [11].

The chemical composition of the powder was investigated by energy dispersive X-ray spectroscopy (EDX, Oxford instruments, Oxford, UK) and inductively coupled plasma optical emission spectrometry (ICP-OES, SPECTRO Analytical Instruments GmbH, Kleve, Germany) to ensure that alloying elements are within specification. The EDX measurement gives an overview of the alloying elements but is relatively inaccurate in the case of light and highly conductive elements like Al and Si. Therefore, an ICP-OES was performed to validate the chemical composition.

For characterization of the powder morphology, the particle size distribution (PSD) was determined with laser diffraction spectroscopy (Mastersizer 2000, Malvern Panalytical Ltd, Malvern, Worcestershire, UK) according to ISO 13320-1 [12] and metallographic imaging by optical and scanning electron microscopy.

For a more reliable assessment of the processed powder, further rheological measurements were performed with the Hall flow test [13] as well as the stability and variable flow test and shear tests with a Freeman Technology-FT4 Powder Rheometer (Freeman Technology, Tewkesbury, UK) corresponding to ASTM D7891-15.

To evaluate the effect of the heat treatment, metallographic investigations via optical and electron microscopy were performed. For the microstructural investigation, the prepared samples were etched with V2A etchant and Kalling 2. For more detailed phase analysis an electron backscatter diffraction (EBSD) investigation was performed at an acceleration voltage of 20 kV, sample tilting of 70°, at a recording speed of 40.33 Hz.

The density measurement was performed in accordance with the Archimedean principle [14] using 11 cuboid specimens with edge lengths of 10 × 10 × 15 mm^3^. The temperature and density of the water were held constant at 22.2 °C and 0.9977 g/cm^3^. In addition, metallographic investigations of the cross sections of three sample cuboids were conducted and analyzed by quantitative image analysis according to [15]. As tensile strength specimen geometry DIN 50, 125—E 3 × 8 × 30 [10] was chosen for reasons of beneficial build-up time, testability, and its representation of the blade geometry of the flexure pivot. The test direction is in the *x*–*y* plane of the build-up. The tensile tests were performed according to [16] at room temperature at an H & P inspect Table 50 kN using test procedure A. The inspection speed was 5 mm/min. The test direction is in the *x*-*y* plane of the build-up.

In addition to the aforementioned destructive tensile tests, the Young’s modulus is also measured by the nondestructive LAwave technique. It is based on the velocity measurement of laser-induced surface acoustic waves in dependence on their frequency to determine elastic properties, density, and thickness of films and surfaces [17]. This method is widely used for the characterization of films and surfaces, i.e., coatings from PVD [18,19,20], or thermal spraying [21,22,23,24].

Surface acoustic waves are elastic vibrations propagating along material surfaces. Their amplitude is highest at the surface and decays exponentially within the material. The velocity of the surface acoustic waves depends on the elastic properties and the density of the material. Furthermore, pores, cracks, segregations, texture, process or hardening layers, and other microstructural properties also influence the wave velocity. The penetration depth of the surface acoustic waves correlates with frequency. Waves with higher frequency propagate closer to the surface. For a completely homogeneous sample the wave velocity is constant for every frequency. The calculation of the wave velocity in dependence on frequency using a theory that contains the materials’ elastic constants and density [25,26] enables the determination of the Young’s modulus. Due to its limited penetration depth, LAwave is not suited for solid volumes. However, it could be applied as a nondestructive test for the flexure pivots since the blade thickness of 200 µm is very small. In addition, comparisons between different heat treatment states could be performed with a single sample.

In this paper, shape deviations were evaluated by a new omnidirectional approach based on 3D scanning and laser scanning microscopy to calculate a reliable offset value for the flexure pivot blades for the postprocessing after the LMD process.

**Figure 7:**
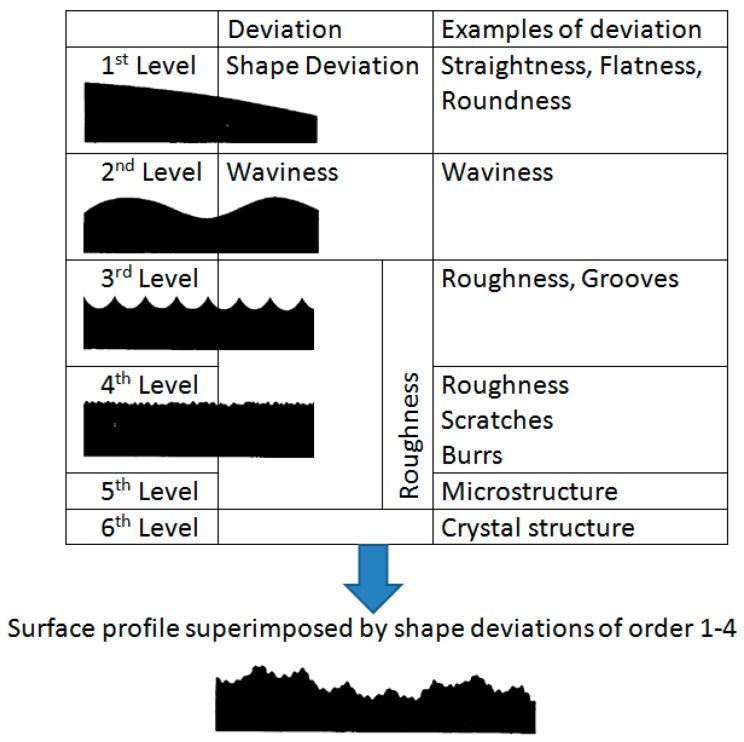
Classification of deviations acc. to DIN 4760 [27].

The surface quality or surface texture is influenced by deviations from the ideal plane on different surface levels classified in DIN 4760 [27]. Possible deviations include shape deviation; waviness, and roughness (see Figure 7).

3D scanning with the GOM Atos Core was used for the omnidirectional measurement of the flatness which represents the minimal distance of two planes that include all points of the surface. The Keyence VK-250 combines laser scanning and confocal microscopy and was used for primary areal and profile surface texture measurements. Scans were taken with a magnification of ten and stitched together resulting in a total area of 12 × 10 mm^2^.

For profile measurements, two directions have been chosen: building direction Z and perpendicular to Z direction X representing the profile along one layer. To increase the accuracy of the measurements, twenty parallel profile lines were evaluated and thus the resulting parameters are averaged values. For areal measurements, a form suppressing filter was used to eliminate the effect of a tilted surface.

The following two profile and areal parameters were measured: the arithmetical mean height S_a_/P_a_ and the maximum height S_z_/P_z_ defined as the sum of the largest peak height value and the largest pit depth value within the defined area/profile [28].

$$S_{a}=\frac{1}{A}\overset{}{\underset{A}{\iint }}\left|z\left(x,y\right)\right|dxdy$$

$$P_{a}=\frac{1}{lr}\int_{0}^{lr}\left|z\left(x\right)\right|dx$$

$$S_{z}=S_{P}+S_{V}=\max_{A}z\left(x,y\right)+\left|\min_{A}\left(z\left(x,y\right)\right)\right|$$

$$P_{z}=P_{P}+P_{V}=\max\left(z\left(x\right)\right)+\left|\min\left(z\left(x\right)\right)\right|$$

### 2.4. Testing Campaign

The main goals of the testing campaign were to characterize the flexible hinge in terms of stiffness and actuation torque as well as to validate the fatigue resistance by means of a lifetime test. The mechanical performance test before the lifetime test was performed over the angular range of ±5°. The lifetime test was performed with the goal of 500,000 cycles in the same angular range of ±5°

## 3. Results and Discussion

### 3.1. Powder Characterization

The EDX measurement gives an overview over the alloying elements but is relatively inaccurate in the case of light and highly conductive elements like Al and Si. Therefore, an ICP-OES was performed to validate the chemical composition. The EDX measurement and the ICP-OES match the chemical composition specified by the powder supplier (Table 3).

**Figure 8:**
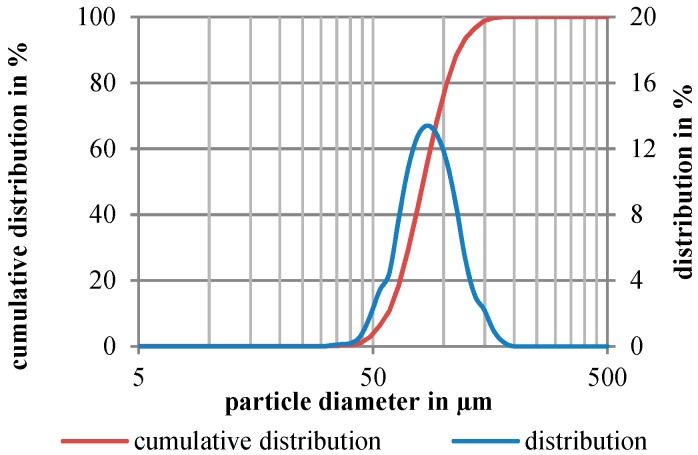
Particle size distribution of the used 316L-Si powder.

For characterization of the powder morphology, the particle size distribution (PSD) shown in Figure 8 was determined with laser diffraction spectroscopy (Mastersizer 2000) according to ISO 13320-1 [12] and metallographic imaging by optical and scanning electron microscopy.

The PSD shows a uniform distribution of the powder fraction with small deviations at the particle diameter 55 and 135 µm, indicating a slightly higher amount of the fine fraction D (v, 0.1) and coarse fraction D (v, 0.9) compared to a completely uniform PSD (Table 4).

The determined volume weighted de Brouckere mean D (4, 3) and area weighted Sauter mean D (3, 2) indicate a uniform distribution of the small and the large particles. The lower value of the Sauter diameter compared to the mean diameter indicates a higher amount of smaller irregular particles in form of satellites with a higher specific particle surface. This presumably affects the flowing behavior of the powder caused by higher interparticular friction.

**Figure 9:**
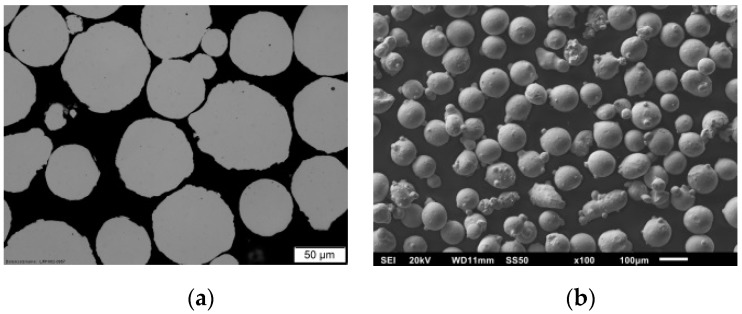
Metallographic images of the 316L-Si powder, (**a**) cutting image, (**b**) SEM image.

The metallographic investigations of the powder shape show that the majority of the particles have a uniform spherical shape (Figure 9). Additionally, some small satellites and irregular shaped particles were detected, confirming the results of the particle size measurements. Overall, friction or interlocking of the particles should only have minor effects on the flowability.

The LMD process depends highly on a uniform and stable powder feed. To ensure a stable feeding behavior, the rheological properties of the powder were investigated. The results of the Hall flow measurements are shown in Table 5.

Significant changes in flow time could not be observed. Hence, the flowing behavior of the powder seems to be constant throughout the batch. Nevertheless, the results of the well-known and established Hall flow test can be affected by the operator. The results of the stability and variable flow rate test and the shear test are shown in Table 6 and Table 7.

The basic flow energy (BFE) of the powder is relatively high compared to steel powders with the equivalent PSD, particle shape, and density, which may be caused by the irregular particle shape and the higher amount of satellite particles. The determined stability index SI of 1.09 indicates a very low tendency to being mobilized. This could be caused by agglomeration, segregation, or electronic charge, which can also be explained by the particle morphology. The powder is insensitive to the flow rate which is indicated by the flow rate index of 1.16. The determined specific energy of the powder of approximately 3.22 indicates a low interparticular cohesivity. Part of the measurement routine is the conditioning of the bulk powder by rotation of the measurement blade to ensure a reproducible quality of the powder sample regarding the bulk density. The measured conditioned bulk density of the powder is 4.34 g/mL, which is equivalent to approximately 55% relative to the bulk density.

For the comparison and a simple classification, two other powders were rheologically characterized: (1) reference spherical 316L-powder with the fraction 50–150 µm and (2) 316L-Si powder with a water moisture content of ~0.1%. In the results of the stability and variable flow rate test for the reference powder, only the values for the BFE and the SE vary. These differences can be explained by the different PSD. Less interparticular contact points lead to less SE, which reduces the BFE.

In comparison to moistened 316L-Si powder, an enormous decrease of the BFE, SI, and CBD was observed. This is attributable to the development of a liquid film between the particles. The film reduces the interparticular friction and increases the tendency of the powder to agglomerate. Due to the worse flowability, the powder also shows a lower bulk density.

In addition, shear tests were performed at ascending levels of stresses for a comprehensive evaluation of the powder flowing (see Table 5). The powder is more cohesive with increasing values of applied normal consolidating stress. In addition, the FF is dependent on consolidating load. Therefore, FF cannot be used as a universal parameter to describe a powder’s flowability alone. The consideration of both static and dynamic rheological tests is necessary to ensure a constant powder feed and sufficient material deposition during the additive process.

Since sufficient knowledge about the correlation between the rheology and the feeding behavior in LMD is not yet available, the powder characterization was concluded by experimental feeding trials. Therefore, a powder feeding rate depending on the feeding speed is determined for both the 316L-Si powder and the reference 316L powder. The derived powder feed lines are shown in Table 7.

**Figure 10:**
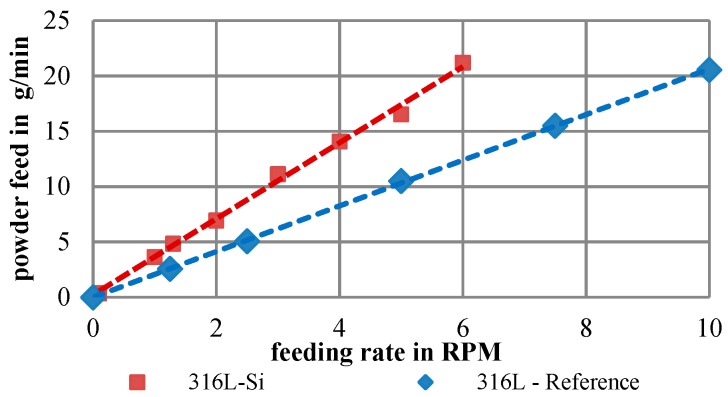
Powder feed line of the investigated 316L-Si and a 316L reference powder.

Despite similar results of rheological properties, apart from slightly deviating specific energies and BFEs (see Table 6), an increased feeding rate of the 316L-Si powder could be observed. The significant differences are caused by the wider particle size distribution. A smaller range provides less interparticle interferences and therefore a better feedability for the process (see Figure 10).

### 3.2. Material Characterization

#### 3.2.1. Metallography

To evaluate the effect of the heat treatment, metallographic investigations via optical and electron microscopy were performed. For the microstructural investigation, the prepared samples were etched with the usual V2A etchant and Kalling 2.

**Figure 11:**
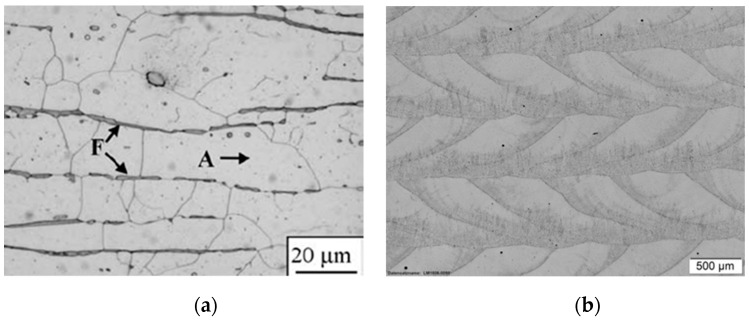
(**a**) Conventional annealed 316L material with ferrite (F) and austenite (A) [29]; (**b**) *x*–*z* cross section of as built 316L-Si sample placed in 45° to the scanning direction.

**Figure 12:**
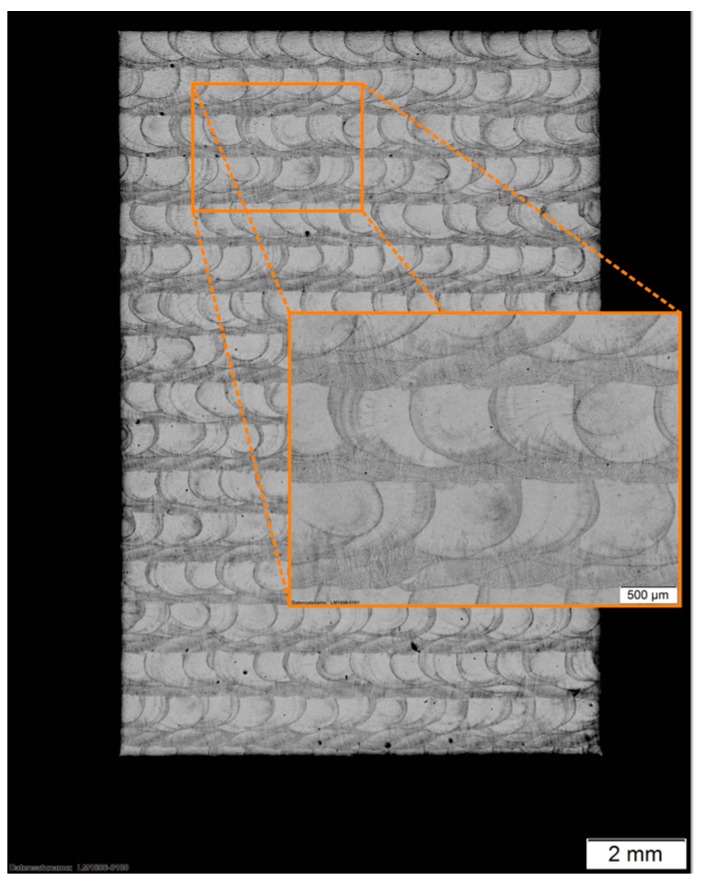
*x*–*z* cross section of heat treated 316L-Si placed 45° to the scanning direction.

**Figure 13:**
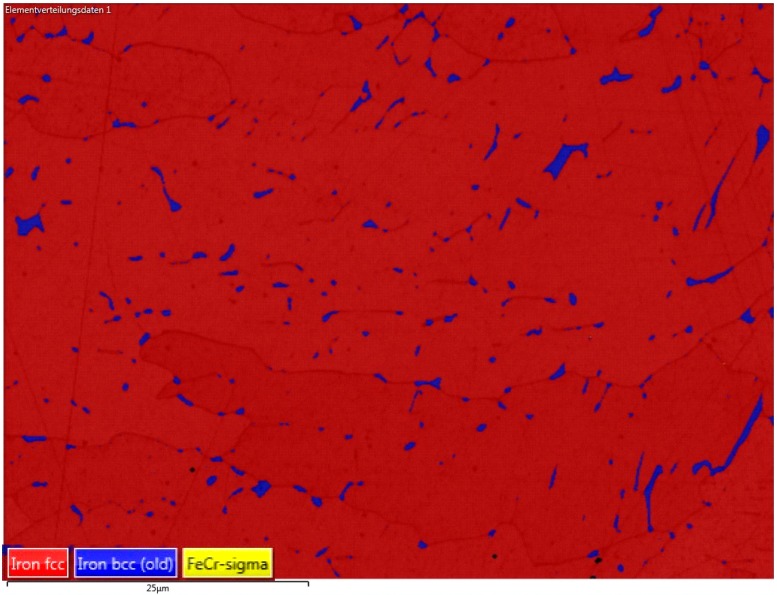
Electron backscatter diffraction (EBSD) mapping at the layer transition zone.

In comparison to bulk material, the x–z cross sections of the as-built (Figure 11) and the stress-relieved sample (Figure 12) show the process immanent typical macrostructure caused by the layer-wise build up and the rotation of the scanning vector by 90° each layer. This macrostructure is not affected by the performed heat treatment. At each transition from layer to layer, a slightly darker area can be observed. By etching with Kalling 2, a high amount of a darker phase at the transition between the layers could be observed. An electron backscatter diffraction (EBSD) investigation shows the phase distribution in this slightly darker area in Figure 13 and the identified phase fractions in Table 8.

**Figure 14:**
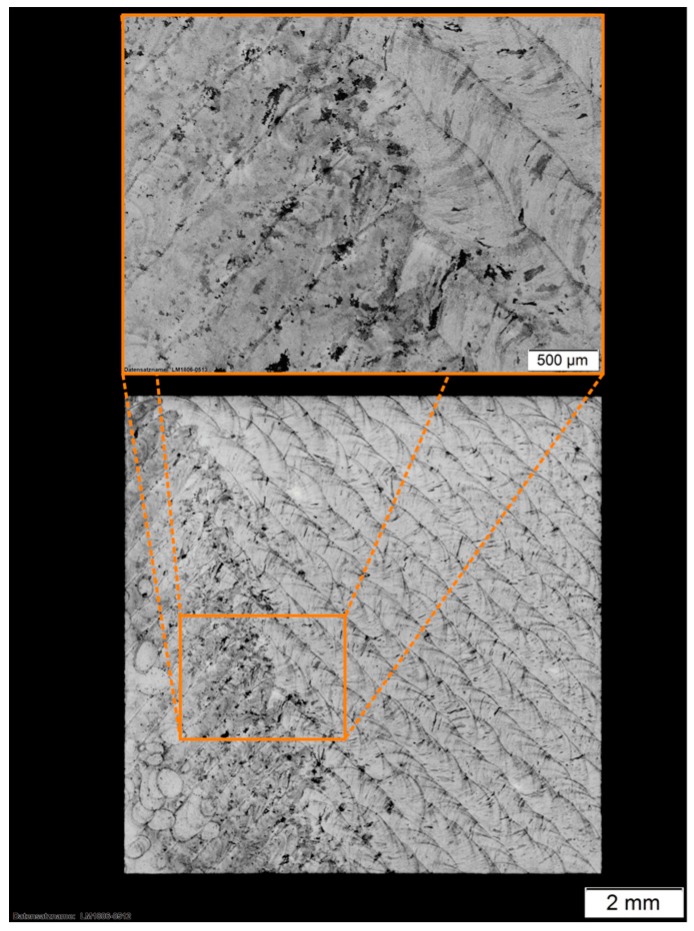
*x*–*y* cross section of the generated and heat treated 316L-Si.

As shown in this investigation, the bcc ferrite phase of 2.6% is distributed inside the fcc austenite phase. The higher amount ferrite phase might be caused by the process immanent partially remelting and heat affection of the subjacent layer, which leads to a transition of the austenite phase into the ferrite phase. The EBSD investigation also shows that there are no undesirable FeCr phases which would also lead to a darkening. In a top view of the generated material, the tracks of two layers are visible (see Figure 14).

**Figure 15:**
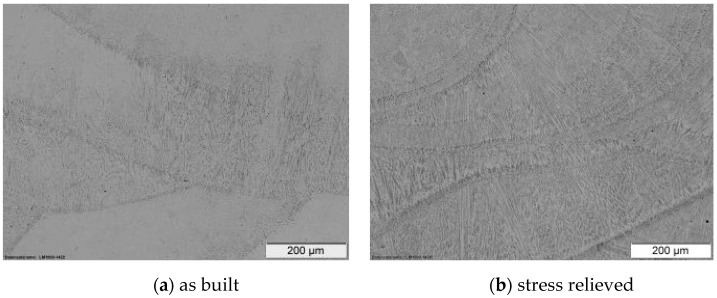
Microstructure of the laser cladded 316L-Si trails (xz-section), (**a**) as built, (**b**) stress relieved.

The main changes in the microstructure in the heat treatment can be observed in the coarsening and homogenization of the ferrite distribution in the white austenite phases (Figure 15). In the as-built sample, the ferrite structures are distributed in the first consolidated areas of each melting track. This is caused by the nonequilibrium consolidation of the melt pool. The annealing leads to a slight coarsening of the microstructure and homogenization of the phases.

**Figure 16:**
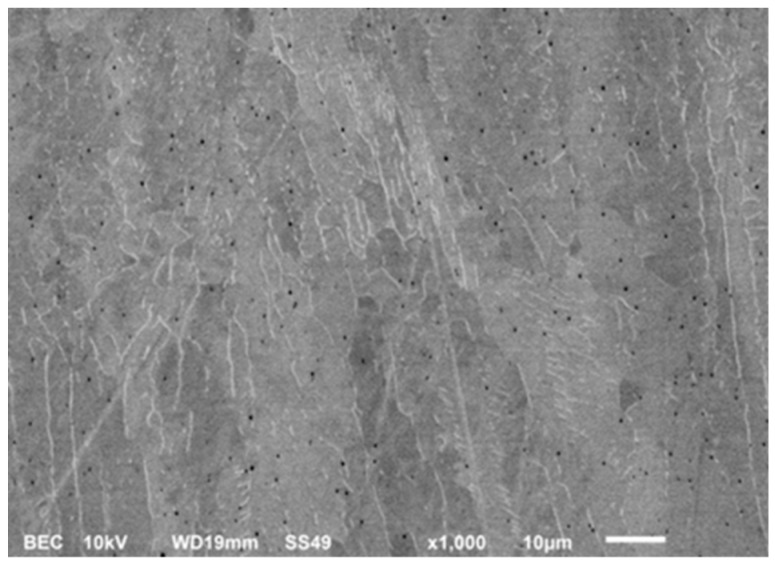
SEM image made with backscattered electron contrast of the as built sample with bright ferrite phases at the grain boundaries, grey austenite grains, and small pores.

The electron microscopic image of the as built sample in Figure 16 also shows the 316L typical austenite and ferrite microstructure with very fine pores.

The metallographic investigations confirm the defect-free build-up and that there is no unwanted grain boundary sensitization due to thermal postprocessing.

#### 3.2.2. Density Test

**Figure 17:**
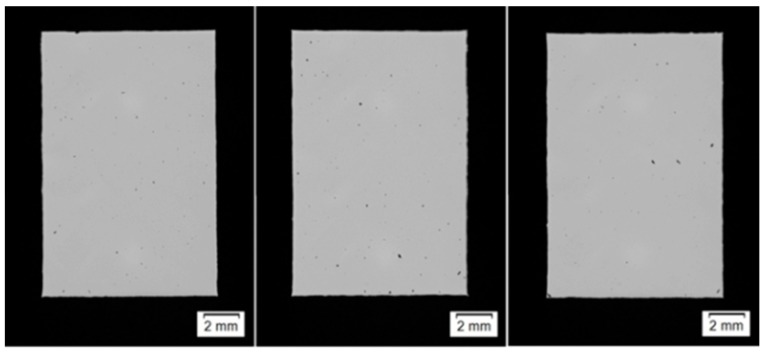
Cross sections of three similarily build up density cubes.

The average density was 7.838 ± 0.003 g/cm^3^, resulting in a relative density of 99.85% compared to the theoretical density of 7.85 g/cm^3^ for 316 L-Si [30]. The investigation of the cross sections of three sample cuboids (see Figure 17) showed an average porosity of 0.15%, which verifies the previous Archimedes test results. The etching of the material shown in Figure 12 indicates that the present pores are located at the edges of the melting tracks, which is usual for the LMD process.

#### 3.2.3. Tensile Test

The tensile strength specimen geometry was chosen for reasons of beneficial build-up time, testability, and its representation of the blade geometry of the flexure pivot. The results for the eight tested specimens are shown in Table 9 and juxtaposed with specified ingot material.

The characterization of the additive manufactured 316 L-Si showed that the yield strength and tensile strength are superior to the standard values of the ingot material, resulting from the fine-grained microstructure caused by the high cooling rates of the powder LMD process. It was also found that the Young’s modulus is similar to the specifications of the ingot.

#### 3.2.4. LAwave

**Figure 18:**
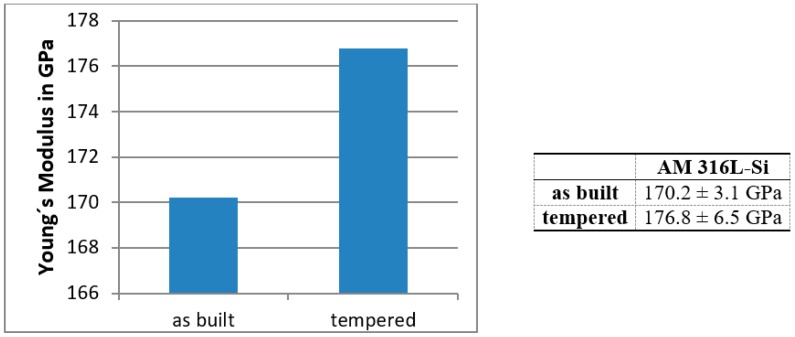
Determined average Young’s modulus for seven specimens.

The Young’s moduli of seven representative 316L-Si blades build by LMD were measured three times each before and after the heat treatment (Figure 18).

Compared to the tensile test and literature results (Table 9), the measured values are slightly lower than expected, which has to be investigated in further research activities. Nevertheless, the new LAwave technique seems to be a promising approach for post-process quality control of additive manufactured metal parts.

#### 3.2.5. Surface Morphology

**Figure 19:**
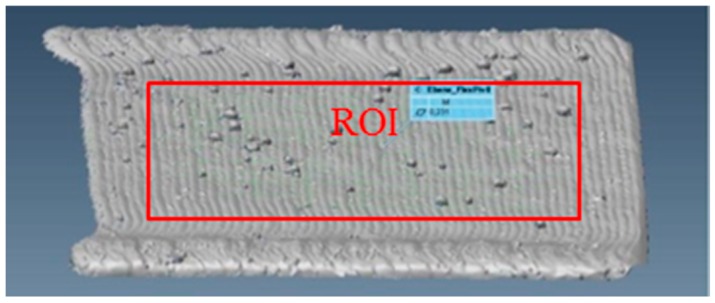
3D scan and region of interest (ROI) to measure the flatness.

To determine characteristic surface parameters for the blades of the flexure pivot, four thin walls were built with the parameter set given in Figure 19. The flatness was measured in the medium section of the plate through 3D scanning. The results are shown in Table 10 and suggest a minimum offset for postprocessing of 300 µm.

**Figure 20:**
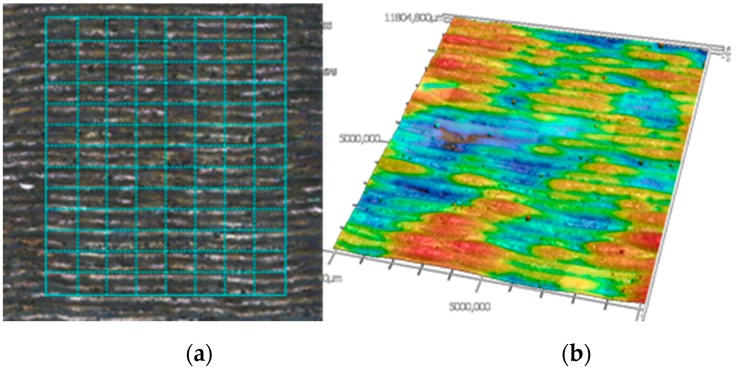
Example of stitching (**a**) and resulting 3D map (**b**) of sample surface 3.

Figure 20 shows the stitched 3D map which was used for the profile and areal measurements.

**Figure 21:**
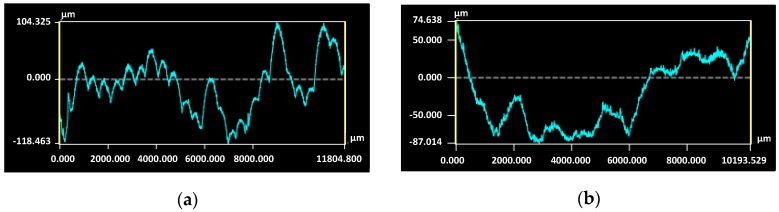
Primary profile of sample 3; (**a**) direction Z, (**b**) direction X.

In Figure 21, the primary profiles of sample 3 in direction Z and direction X are shown. Each of the local peaks visible in direction Z represents one layer.

In Table 11, the profile and areal parameters derived from the laser scanning microscope measurements are shown.

Both the arithmetical mean height P_a_ and the maximum height P_z_ of the primary profile measurements in direction X were consistently higher than in direction Z. The standard deviation of P_z_ in the Z direction is very low with 2.9% and shows a good repeatability. The values for the areal parameter S_a_ are similar to the results of P_a_ in direction X, whereas the maximum height S_z_ is significantly higher than P_z_ in both directions. This is due to the fact that profile peaks or valleys almost never include areal peaks/valleys [31]. Therefore, the areal parameter S_z_ is better suited than profile parameters to derive a necessary offset for postprocessing. Compared to the flatness values obtained by 3D scanning, measured values for S_z_ are higher. Thus, the machining offset for this application should be at least 350 µm.

### 3.3. Redesign and Postprocessing

**Figure 22:**
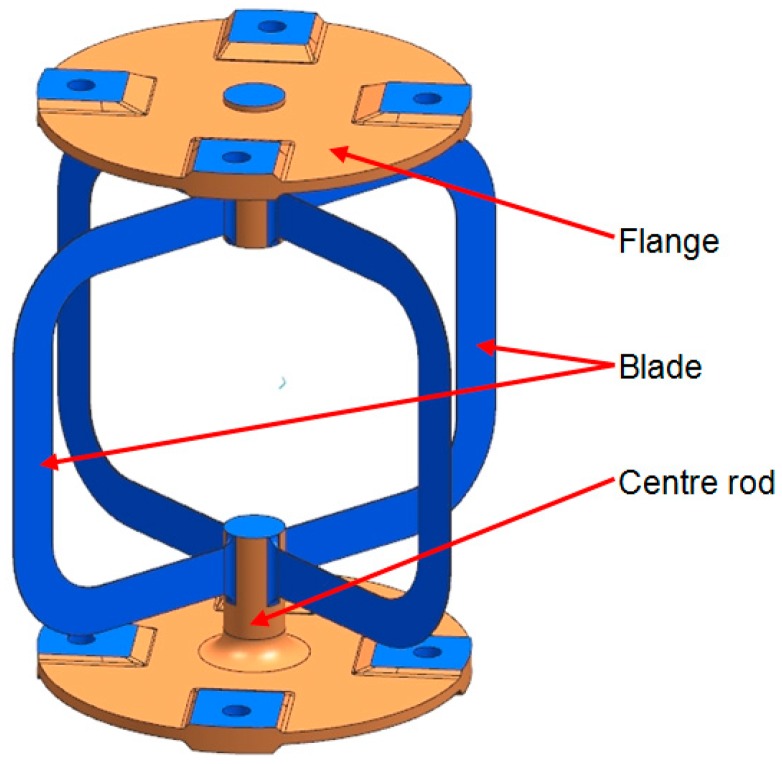
AM flexible pivot with functional elements (postprocessed surfaces are highlighted blue).

The obtained data from the material characterization such as yield strength, tensile strength, and surface quality are directly incorporated in the redesign of the flexure pivot. Based on the conventional design, the aforementioned LMD characteristics and constraints were considered as well (Figure 22). The main functional elements of the flexure hinge are the center rod, the flanges, and the four blades.

**Figure 23:**
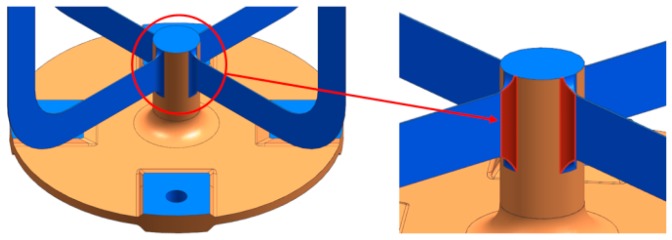
Intersection radius from blade to center rod.

The design is mainly driven by the manufacturing concept, its characteristics, and the proposed postmachining. It is essential to ensure full access for the traverse paths of the laser and machining heads. This influences, for example, the dimensioning of the radii at the intersection from the blades to the rod, which are shown in Figure 23.

**Figure 24:**
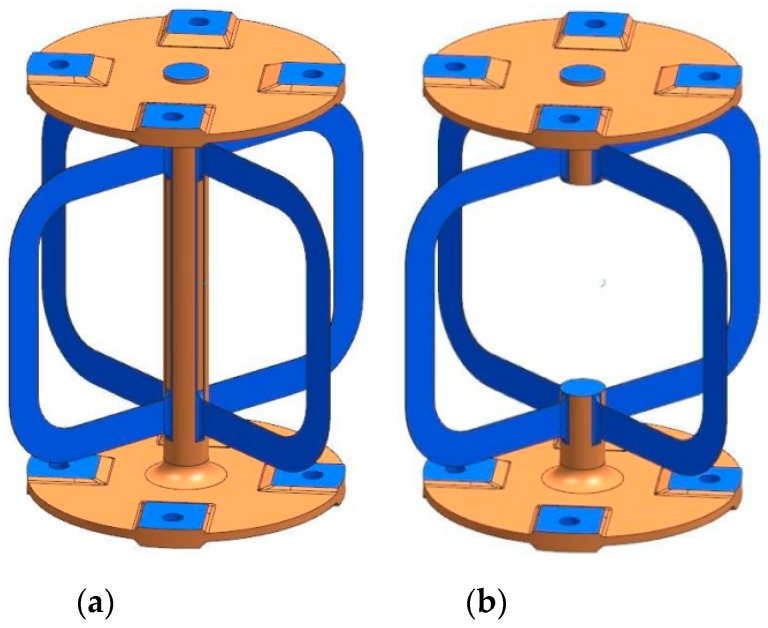
(**a**) Semifinished hinge with center rod; (**b**) final hinge with detached rod.

The 316L center rod functions as substrate as well as a support during the manufacturing process. Consequently, the main loads during postprocessing are carried by the rod. Therefore, the detachment of the center rod is the last manufacturing step (see Figure 24).

The flanges connect the satellite with the optical element (e.g., mirror or antenna). The four-hole pattern at the flanges enables mounting of the flexible hinge and introduces rotational torque. Both flanges are identical and mirrored through the median plane of the pivot. The overall wall thickness is 2 mm with thickenings up to 4.5 mm for the mounting surfaces.

The most challenging elements of the flexure hinge are the blades which are highly loaded and therefore require accurate manufacturing. The blades have a wall thickness of 0.25 mm with a height of 8 mm. Four identical blades are positioned in a 90° pattern around the center rod, providing the flexible elements of the flexure pivot with low bending and torsion stiffness due to the low wall thickness and rectangular cross-section.

**Figure 25:**
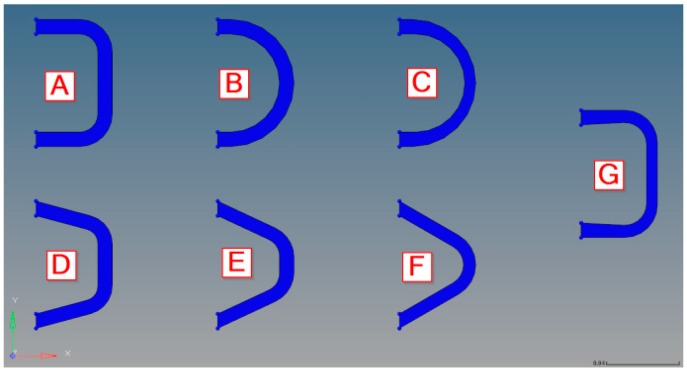
Analyzed blade shape designs.

Several different shapes of the blade have been analyzed regarding the rotational stiffness, maximum stress, and parasitic shortening of the hinge at 10 and 30° deflection (see Figure 25). The analysis was performed on a reduced model of one single blade of each contour.

The results of these analyses are shown in Table 12.

For the optimal functioning of the flexure hinge, a compromise between low actuation torque, low stress values, and small parasitic shorting is needed. Considering these criteria, concept A was chosen for the manufacturing design.

**Figure 26:**
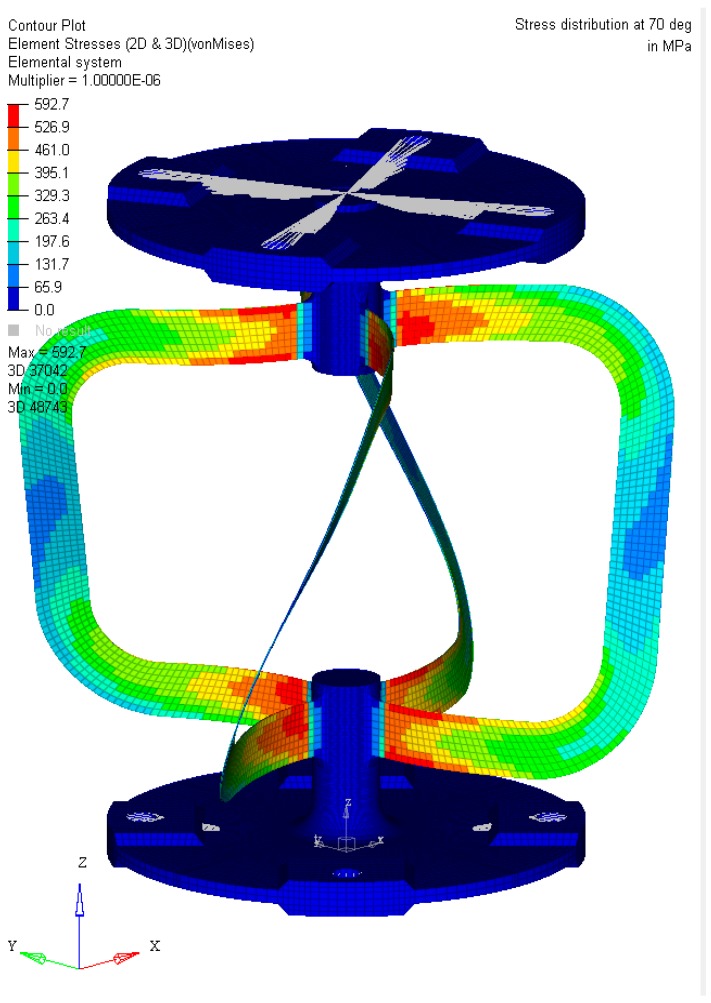
Overview of stress at the flexure hinge by 70°.

**Figure 27:**
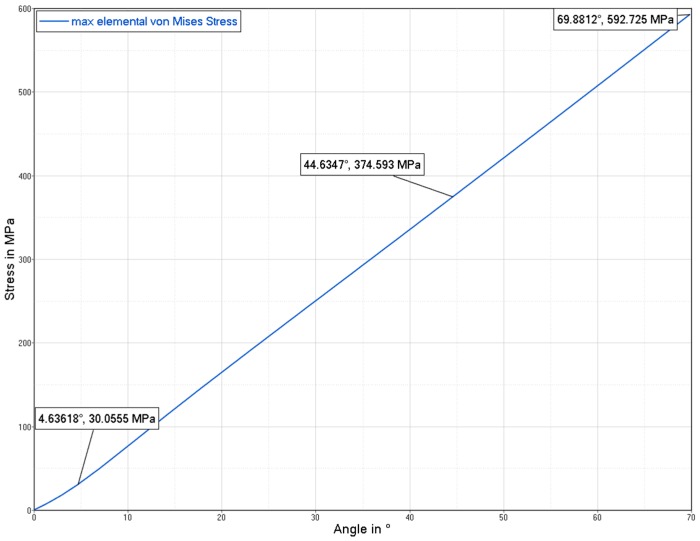
Maximum stress vs. hinge deflection.

According to the material parameters provided in the previous chapter, a finite element model of the flexure hinge was created with Hypermesh 17.2 in order to perform a nonlinear analysis using Optistruct (Nastran clone) (see Figure 26 and Figure 27). The boundary conditions were set as follows:

- Fixed constraint (all degrees of freedom blocked) at the bottom flange (fix interface).
- Enforced rotation on the top flange (input interface) around the *z*-axis. Since the model was created to be as symmetrical as possible and as the analysis shows minimal center shift, no further constraints in the *x*–*y* plane were necessary at the input interface.

In future research activities, the functionality, performance, and lifetime will be tested.

### 3.4. Manufacturing of the Flexible Pivot

**Figure 28:**
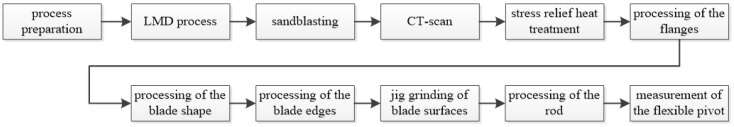
Manufacturing workflow.

The functionality and durability of the flexible pivot places extremely high demands on the entire production chain, which is illustrated in Figure 28.

**Figure 29:**
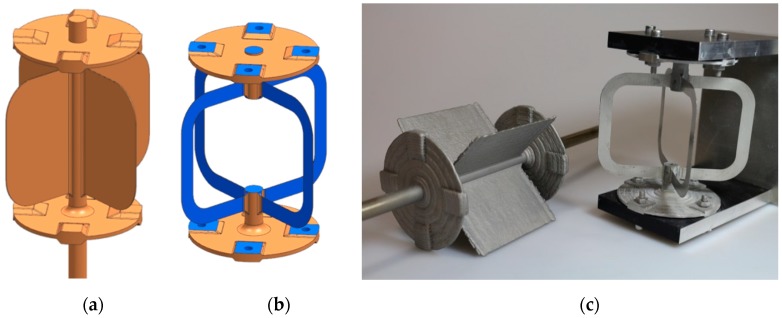
(**a**) CAD model AM part, (**b**) CAD model flexure pivot, (**c**) generated semifinished part with LMD (left) and final flexure pivot for demonstrator testing (right).

Starting with the build-up of the complex part by powder LMD, including several demanding five-axis welding strategies, surficial cleaning by sandblasting was conducted. After a computer tomography scan for quality assurance, a stress relief heat treatment at 420 °C with a retention time of one hour under an Ar atmosphere and a furnace cooling were applied. The subsequent postmachining of the blade geometry includes turning and drilling of the mounting surfaces and wire electrical discharge machining (wire-EDM) followed by jig grinding of the blades to reduce the blade thickness from 1.4 mm down to 0.25 mm, which proved to be very challenging. The filigree shape and thickness of the blades yield vibration and bending due to machining forces, leading to poor surface quality or even tearing off of the blades. As a countermeasure, a special clamping device was designed and manufactured providing a lay-on support that absorbs the mechanical forces during the grinding process. Figure 29 shows the transition from the as-built AM part to the operational flexible pivot.

### 3.5. Demonstrator Testing Campaign

The stiffness in the X and Y directions was in the range of +10/−21% of the test predictions. In the Z direction, the demonstrator was about 45% softer than expected. The measured rotational stiffness was also about 30% softer than the predictions. The deviations are in an arguable range due to the different achieved thicknesses of the blades compared to the calculated model with blades with constant thickness.

**Figure 30:**
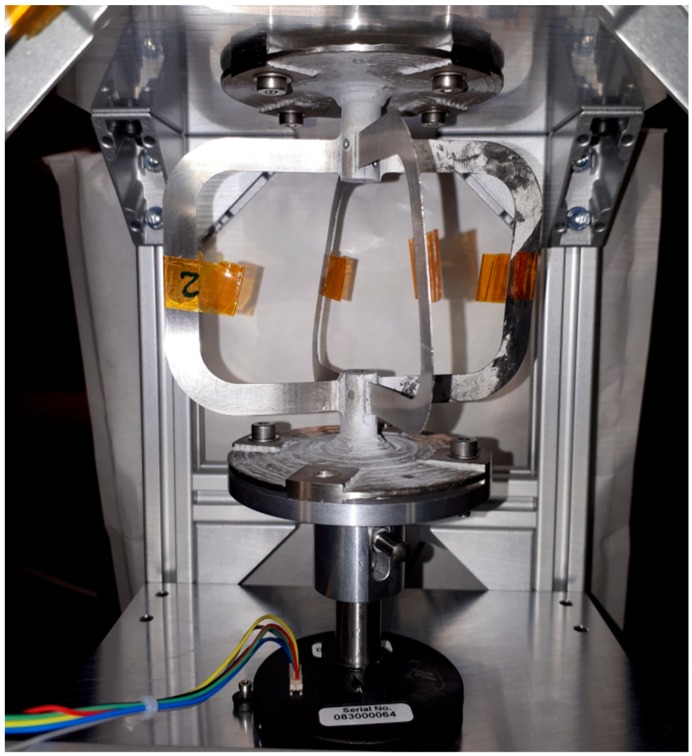
Flexure hinge integrated in the lifetime test bench.

The lifetime test was performed with the goal of 500,000 cycles in the same angular range of ±5° and resulted in no deformation or damage of the demonstrator. The extreme values of the torque over the complete lifetime exceeded the required range, but these deviations were caused by a small noise of the measured signal influenced by the electrical field of the engine (see Figure 30).

The performance test at end-of-life (EOL) over the range of ±5° showed no significant degradation of the rotational stiffness (from 0.21 Nm/rad to 0.19 Nm/rad). Even the performance test EOL over the range of ±30° did not affect the stiffness. The rotational stiffness throughout the tests was approximately 30% softer than the test prediction (see Table 13).

In summary, the demonstrator test campaign was successful. The lifetime was reached at ±5° and throughout the whole test campaign, the demonstrator suffered no damage.

## 4. Conclusions and Outlook

During the concept design process of the flexure pivot, potential manufacturing challenges, such as the surface quality and geometrical accuracy, were identified. Mechanical damages induced by stress concentrations related to surface imperfections or high surface roughness increase the risk of premature failure. Therefore, the need for a combined additive and subtractive process approach was identified.

For the additive process, LMD was chosen because no support structures are needed and complex geometrical features can be added to semifinished parts, which reduces manufacturing time and potentiates the advantages of hybrid AM processes.

Within the paper, the interconnection of the results from comprehensive powder characterization, metallographic investigation, material testing, and demonstrator testing provides high transparency of the powder LMD process, which is crucial in space applications. The main flow properties, the morphology, and chemical composition of the 316L-Si powder have been identified. It was found that high flow energy correlates to a high bulk density, low specific energy, and short average flowtime, and uniform spherical particle shape reflects a low degree of cohesion. The latter properties support adequate performance in terms of process stability and reproducibility. Furthermore, it can be stated that:

1. The fraction of the powder should be kept narrow to reduce interparticle interference and
2. The humidity of the powder has a huge impact on the processability. To avoid obstructions in the process and to ensure the quality of the flexure pivot it is recommended to dry the powder before processing.

Furthermore, advanced approaches in rheological powder characterization, omnidirectional roughness, and nondestructive Young’s modulus measurements yielded outstanding advantages for quality control of additive manufactured components.

The biggest challenge during the development process was to optimize the design and manufacturing plan of the flexure hinge to meet the required quality and accuracy. The postprocessing of the blades to the required wall thickness proved to be time consuming and costly. The flexure hinge was successfully manufactured and the scheduled lifetime was reached in a test campaign, proving that additive manufacturing can produce functional parts.

Furthermore, in space applications, a big potential of additive manufacturing is seen in functional integration of, for example, electrical components or functional tribological surfaces with reduced postprocessing steps.

## Figures and Tables

**Table 1:** LMD machine setup.

Machine	Hermle C20 U
Laser	Laserline LDF 1500-400
Laser Wavelength	915–940 nm
Spot-ø	1.6 mm
Nozzle	COAX14 (working distance: 7.5 mm)
Powder Feeder	GTV PF 2/2
Powder	Oerlikon 316 L Si (44–106 µm)
Feeding/Shielding Gas	Argon 5.0
Substrate	stainless steel (1.4301) with 10 mm thickness

**Table 2:** Material sample parameter set.

Process Parameter	Value
Laser Power in W	550
Laser Scanning Speed in mm/min	600
Powder Feed Rate in g/min	4.16
Laser Spot Diameter in mm	1.6
Track Overlap in %	50
Single Track Width in mm	1.00
Layer Thickness in mm	0.91

**Table 3:** Chemical composition of AISI 316L-Si powder (EDX) compared to chemical composition provided by supplier [11] and standard.

Mass Fraction in %	Fe	Cr	Ni	Mo	Si	Mn
Data Sheet	rest	17.0	12.0	2.5	2.3	1.0
Standard	rest	16.0–18.0	10.0–14.0	2.0–3.0	<1.0	<2.0
EDX	64.5	18.0	11.3	3.3	2.9	0.1
ICP-OES	65.2	17.5	12.2	2.6	2.2	0.3

**Table 4:** Mean representative particle diameters including the Sauter mean, the de Brouckere mean and the percentile means.

Span	D (4, 3) in µm	D (3, 2) in µm	D (v, 0.9) in µm	D (v, 0.1) in µm	D (v, 0.5) in µm
0.71	85.15	78.91	115.76	57.94	82.05

**Table 5:** Results of Hall flow test [13].

Test Number	1	2	3	Ø
**Flow Time in s**	23.41	23.76	24.42	23.86

**Table 6:** Results of rheological test of 316L-Si compared to a wet and a reference 316L powder.

Test Number	BFE in mJ	SI	FRI	SE in mJ/g	CBD in g/mL
1	773.26	1.20	1.26	3.02	4.36
2	846.54	1.16	1.21	3.27	4.33
3	886.38	1.12	1.16	3.41	4.30
Ø	821.38	1.09	1.16	3.22	4.34
Wet	372.65	0.66	1.12	3.95	2.50
Ref.	658.60	1.06	1.12	2.37	4.38

BFE: Basic flow energy; SI: Stability index; FRI: Flow rate index; SE: Specific energy; CBD: Conditioned bulk density.

**Table 7:** Results of the shear tests for Metcoclad 316L-Si.

Shear	C in kPa	UYS in kPa	MPS in kPa	FF	AIF in °	BD in g/mL
3.00	0.28	0.84	4.37	5.18	22.41	4.65
6.00	0.33	0.96	7.75	8.09	21.67	4.48
9.00	0.48	1.42	12.05	8.50	21.48	4.54
15.00	0.54	1.59	19.73	12.41	21.89	4.45

C: Cohesion; UYS: Unconfined yield stress; MPS: Mayor principle stress; FF: Flow function; AIF: Angle of internal friction; BD: Bulk density.

**Table 8:** Identified tabled phase fractions.

Phase Name	Phase Fraction in %
Iron fcc	97.38
Iron bcc (old)	2.60
FeCr-sigma	0.00

**Table 9:** Average material properties of the heat treated 316 L-Si specimens and the material specifications according to [30].

Material	AM 316L-Si	Ingot 316L-Si
R_p0,2_ in MPa	451 ± 6	>170
R_m_ in MPa	693 ± 2	>485
E in GPa	189 ± 7	190–210
A_g_ in %	38.0 ± 1	>30
A in %	50.0 ± 1	>50

R_p0,2_: yield strength; Rm: tensile strength; E: Young’s modulus; Ag: uniform strain; A: elongation at break.

**Table 10:** Flatness of surface measured with 3D scanning.

Sample	1	2	3	4	Mean ± Std. Dev.
Flatness in µm	293	278	257	221	262.25 ± 27.03 (10.31%)

**Table 11:** Primary profile and areal parameters of the sample surfaces measured with laser scanning microscopy.

Sample	X Direction		Z Direction		Area	
	P_a_ in µm	P_z_ in µm	P_a_ in µm	P_z_ in µm	S_a_ in µm	S_z_ in µm
Mean value	46.23	246.35	36.86	178.67	44.11	350.62
± std. dev.	8.91%	7.61%	7.30%	2.90%	5.13%	3.65%

**Table 12:** Rotational torque, stresses, and parasitic shortening at one blade by 10 and 30° deflection.

Concept Option		Actuation Torque in Nm		Max. Von Mises Stresses in MPa		Shorting in mm	
		10°	30°	10°	30°	10°	30°
A	C section	0.04	0.08	69.9	266.3	0.12	1.17
B	tube section	0.05	0.09	87.9	284.2	0.14	0.97
C	tapered tube section	0.03	0.08	63.1	262.3	0.13	1.24
D	large trapezoidal section	0.05	0.09	82.2	290.2	0.15	1.20
E	mid trapezoidal section	0.05	0.11	107.9	336.5	0.14	1.15
F	small trapezoidal section	0.05	0.10	113.9	335.9	0.14	1.03
G	tapered C section	0.02	0.06	56.4	226.9	0.14	1.33

**Table 13:** Main stiffness characteristics of lifetime setup at beginning of life (average of three measurements).

Rotational Stiffness Around Center axis	0.21 Nm/rad
Tensile Stiffness in *x* Direction (lateral 1)	1.02 × 10^4^ N/m
Tensile Stiffness in *y* Direction (lateral 2)	7.24 × 10^4^ N/m
Tensile Stiffness in *z* Direction (axial)	1.23 × 10^5^ N/m
